# Supplementary material for: First come, first served: superinfection exclusion in Deformed wing virus is dependent upon sequence identity and not the order of virus acquisition
Source: ISME J. 2021 Jun 30;15(12):3704–13. doi: 10.1038/s41396-021-01043-4 (PMC8630095; doi:10.1038/s41396-021-01043-4)
Supplement: Supplementary file 1 — Supplementary materials [file 41396_2021_1043_MOESM1_ESM.pdf]

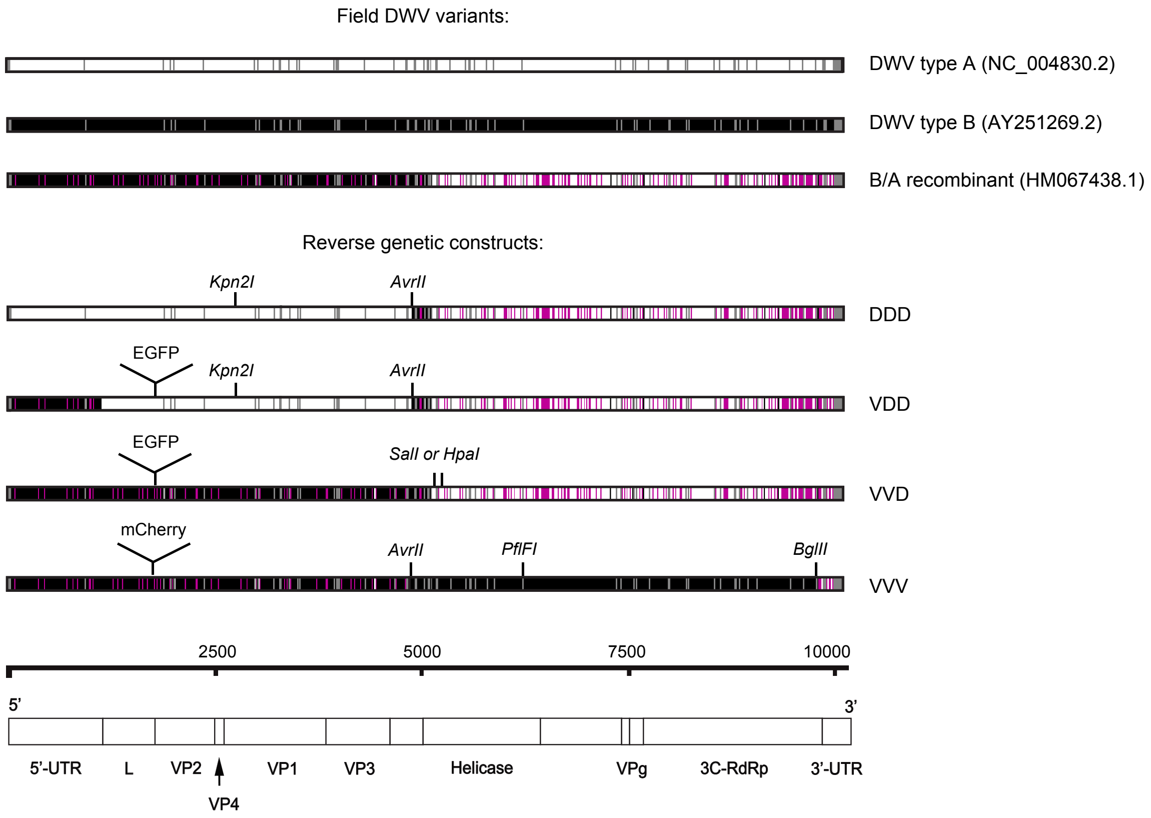


**Figure S1**. **Modular reverse genetics system design for DWV.** Diagram showing homologous parts between genomic RNA sequences of field DWV type A and B variants and the recombinant B/A clone used as a backbone for RG construction. DWV A and DWV B specific sequences are shown in white and black respectively, sequences unique to VDV-1-DWV-No-9 (GenBank HM067438.1) recombinant are shown in purple, identical regions between all three variants are shown in grey; positions of the new restriction sites introduced into each RG construct as genetic markers and location of reporter gene inserts are marked above the construct sequences; DWV genomic RNA organization is shown at the bottom: L - leader protein, VP1-VP4 - capsid proteins, VPg - virus protein genome-linked, 3C-RdRp - 3C protease, and RNA-dependent RNA polymerase complex, 5' and 3'-UTR - flanking untranslated regions. VDD, VVD, VVV, and DDD indicate constructed genomes based upon the modular structure of the DWV genome. Where fluorescent reporter-expressing derivatives were made (see [25] for indicative construction details) the name is suffixed with a subscripted E for EGFP-expressing or mC for mCherry expressing genomes, hence $VVD_E$ or $VVV_{mC}$. VVD variant cDNA was constructed in two versions – either containing *SalI* or *HpaI* restriction site as a genetic 'tag'; these were further designated as $VVD_S$ and $VVD_H$ respectively.

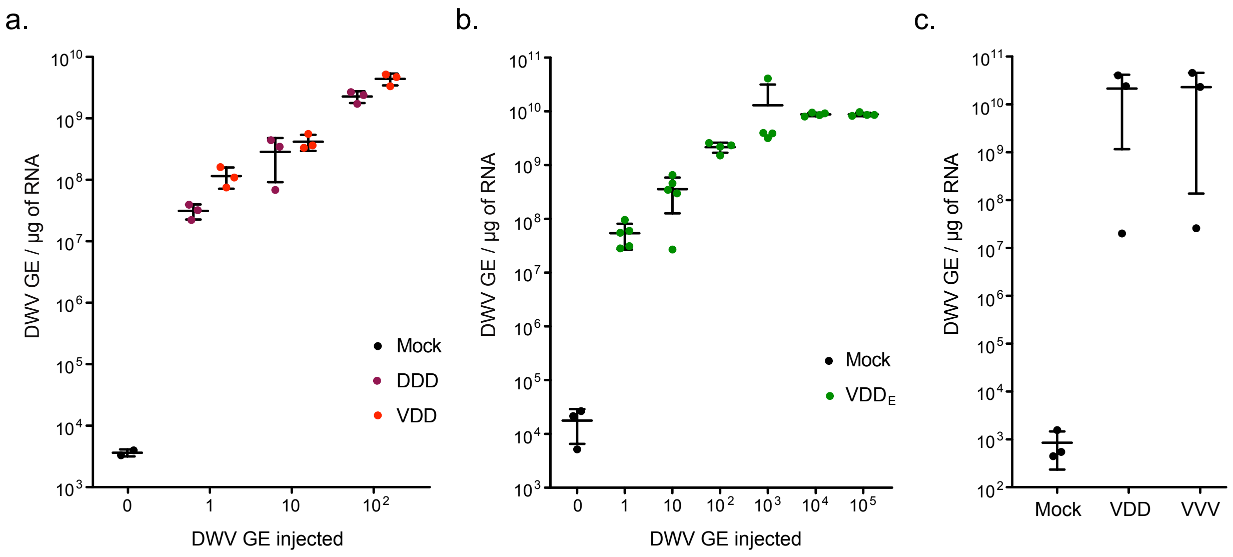


**Figure S2. qPCR analysis of DWV accumulation in honey bee pupae.** Pupae were inoculated with DWV via injection at white-eyed pupal stage or via feeding at larval stage. When injected pupae were analysed at 24 h post-inoculation. Dots correspond to the virus level in individual samples, error bars show mean ±SD, GE - genome equivalents, “Mock” – non-inoculated pupae. **a.** Level of DWV in pupae injected with DDD or VDD virus. **b.** Level of DWV in pupae injected with VDD$_E$ virus. **c.** DWV accumulation in honey bee pupae developed from larvae orally infected with $10^7$ GE of VDD or VVV DWV. Viral titres were quantified on day 8 post-infection.

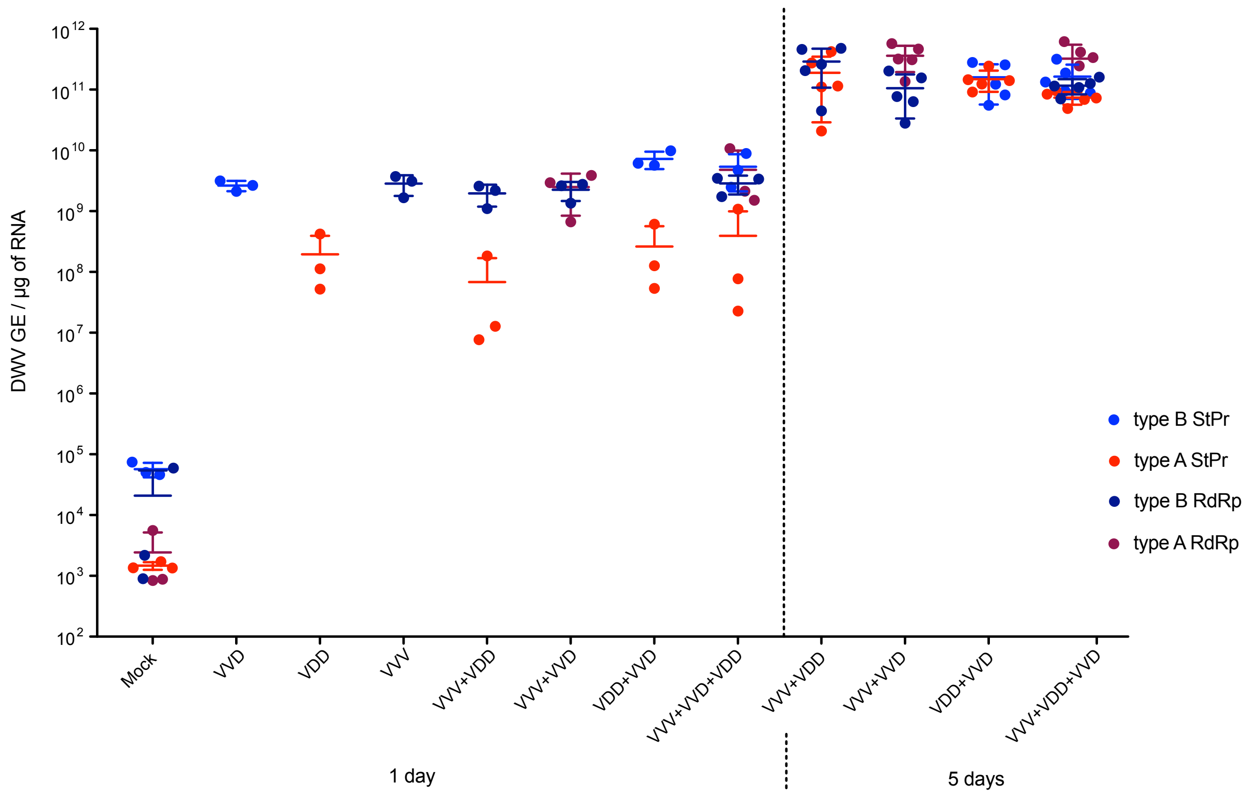


**Figure S3**. **Time course analysis of accumulation of DWV variants in honey bee pupae.** Mock (no injection) and virus-injected pupae were analysed 1 day and 5 days post-injection. Total amount of DWV administered per pupa was $10^2$ GE. Levels of specific genomic RNA were quantified individually in each sample using primer sets targeted to polymerase encoding region (RdRp primers) or structural proteins encoding region (StPr primers) of DWV type A and B. A StPr and B RdRp primers are specific to VDD and VVV DWV variants respectively, A RdRp primers target DWV type A cDNA region identical between VVD and VDD virus clones, B StPr primers target cDNA region identical between VVV and VVD variants. Each data point corresponds to an individual sample, in the case of mixed infection two or four (for three-component infection) data points indicating levels of each target are shown for each sample in the group. Error bars show the mean level of each sequence variant quantified in the injected group ±SD, GE - genome equivalents.

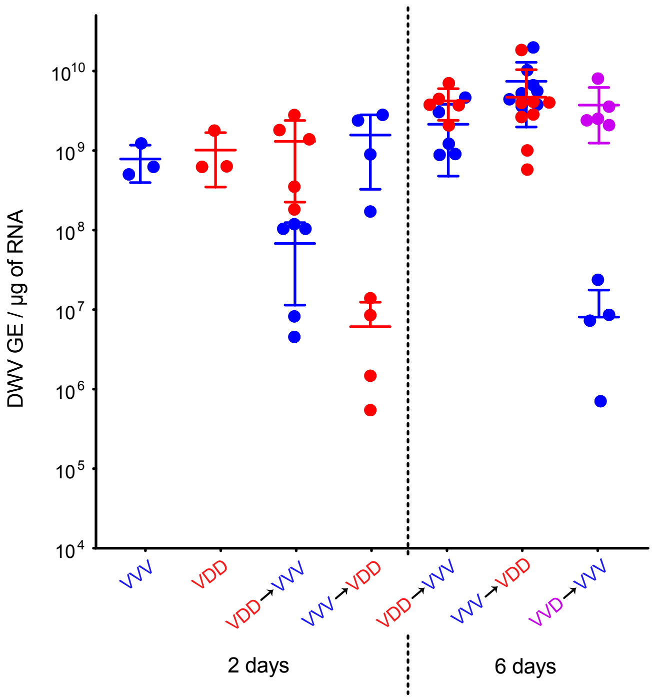


**Figure S4**. **Accumulation of DWV variants in superinfected bumble bee pupae.** Bumble bee pupae were inoculated with one variant of DWV (VVV, VDD or VVD; $10^3$ GE per pupa), then left for 2 days before being inoculated with a second variant ($10^6$ GE per pupa). Individual pupae were analysed after 2 or 6 days of incubation after superinfection by qPCR with type-specific primer pairs. Data points represent DWV levels in individual samples with two points of different colour corresponding to different virus variants (red for VDD, blue for VVV, and purple for VVD respectively) in the same pupa (or in individual pupae for VVV, VDD or VVD only injected samples). Error bars show mean ±SD for each virus variant in each injection group, GE - genome equivalents.

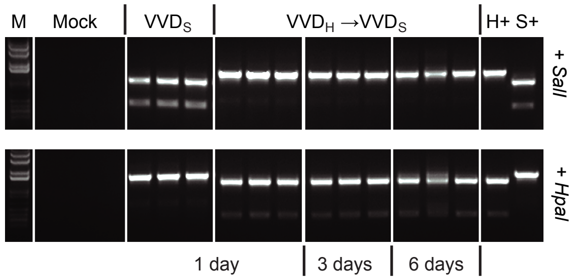


**Figure S5**. **Detection of DWV RNA with different restriction site tags by RT-PCR**. 1% agarose gel of restriction digest products shown. PCR samples in the upper panel were digested with *SalI*, those in the lower panel with *HpaI*. M - molecular size DNA marker, Mock - non-injected pupae samples, "VVD$_S$" – pupae, injected only with VVD virus tagged with *SalI* site (analysed 1 day after infection); "VVD$_H$→VVD$_S$" – pupae injected with VVD$_H$ (*HpaI* tagged VVD DWV) and superinfected with VVD$_S$ (*SalI* tagged VVD DWV) 1 day after the first injection and analysed at 1, 3, and 6 days after superinfection. S+ and H+ are positive PCR controls for products containing *SalI* and *HpaI* sites respectively.

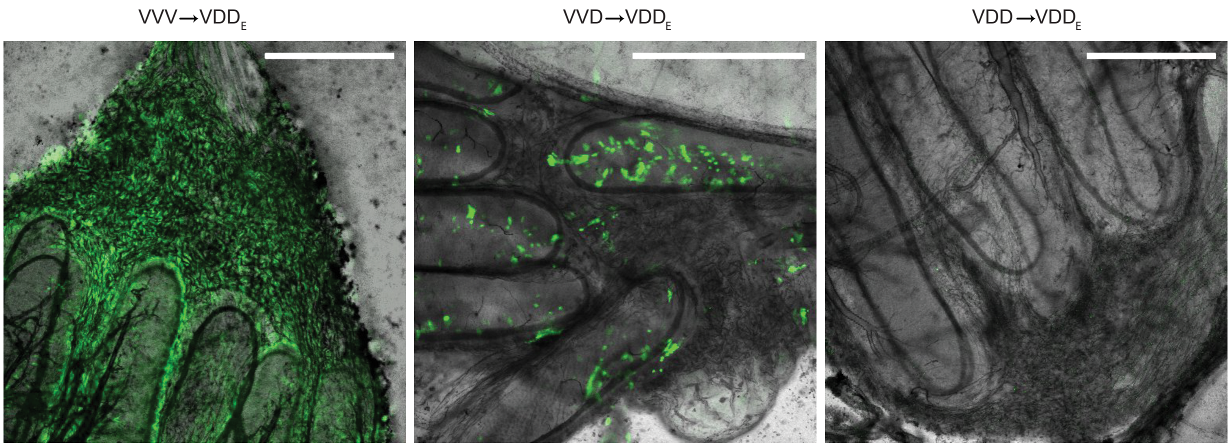


**Figure S6. Confocal microscopy analysis of dissected honey bee pupae injected with different DWV variants and superinfected with VDD$_E$.** Combined white-field and fluorescent images for rectum tissue of infected pupae are shown as examples. Pupae were analysed 6 days after the second injection. Scale bars correspond to 500 μm.

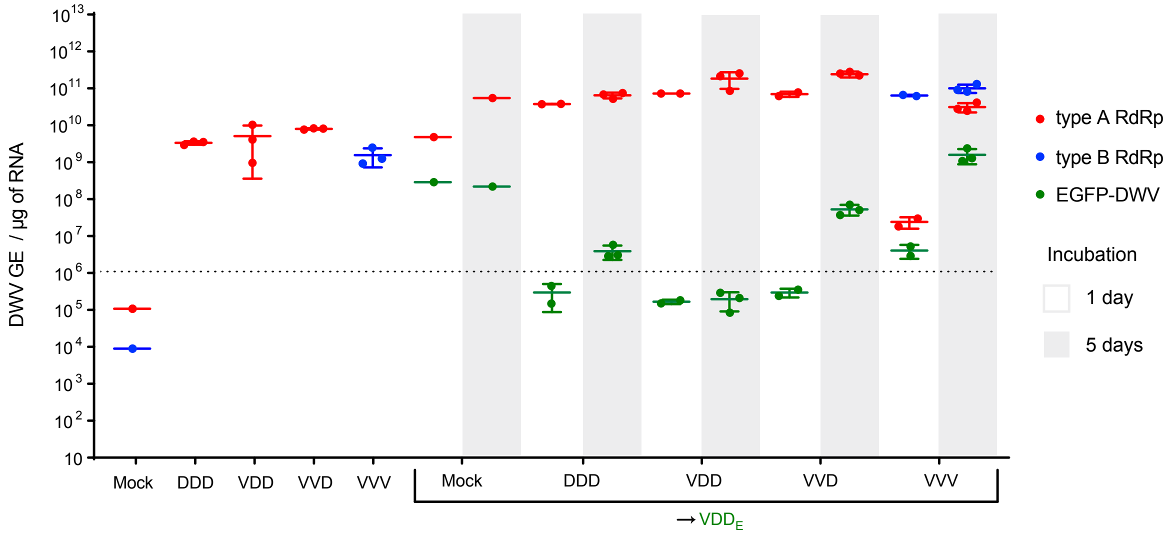


**Figure S7. Time-course analysis of DWV variants accumulation in honey bee pupae superinfected with VDD$_E$.** Pupae were analysed 1 and 5 days after the last injection. qPCR analysis of the virus load was performed using primer sets targeting specific sequences encoding RNA polymerase of DWV type A (red dots) and type B (blue dots). Specific EGFP-encoding DWV RNA was detected by a primer pair targeting the junction between the EGFP insert and DWV genomic sequence (green dots). Each data point corresponds to an individual sample, in the case of mixed infection two or three data points indicating levels of each target sequence are shown for each sample in the group. Lines show the mean level of each sequence in the injected group ±SD, GE - genome equivalents. Individual pupae with VDD$_E$ RNA levels above the threshold indicated with the dotted line had visible EGFP signal when analysed by microscopy.

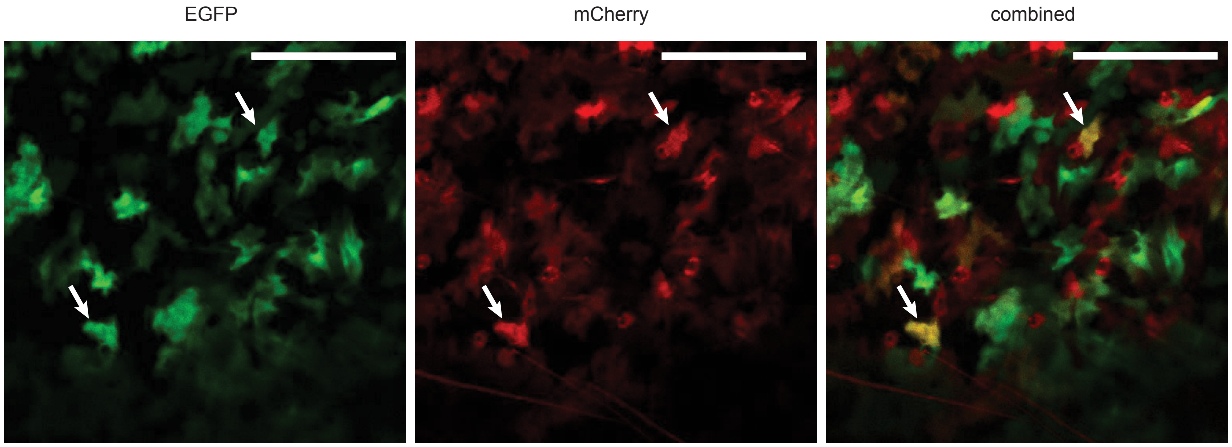


**Figure S8. Confocal microscopy analysis of a dissected honey bee pupa coinfected with DWV variants encoding EGFP ($VDD_E$) and mCherry ($VVV_{mC}$).** EGFP, mCherry, and a combined image for both fluorophores obtained from a section of a digestive tract tissue are shown. Pupae were analysed 3 days after injection. Individual foci expressing both fluorophores are indicated by arrows. Scale bars correspond to 150 μm.

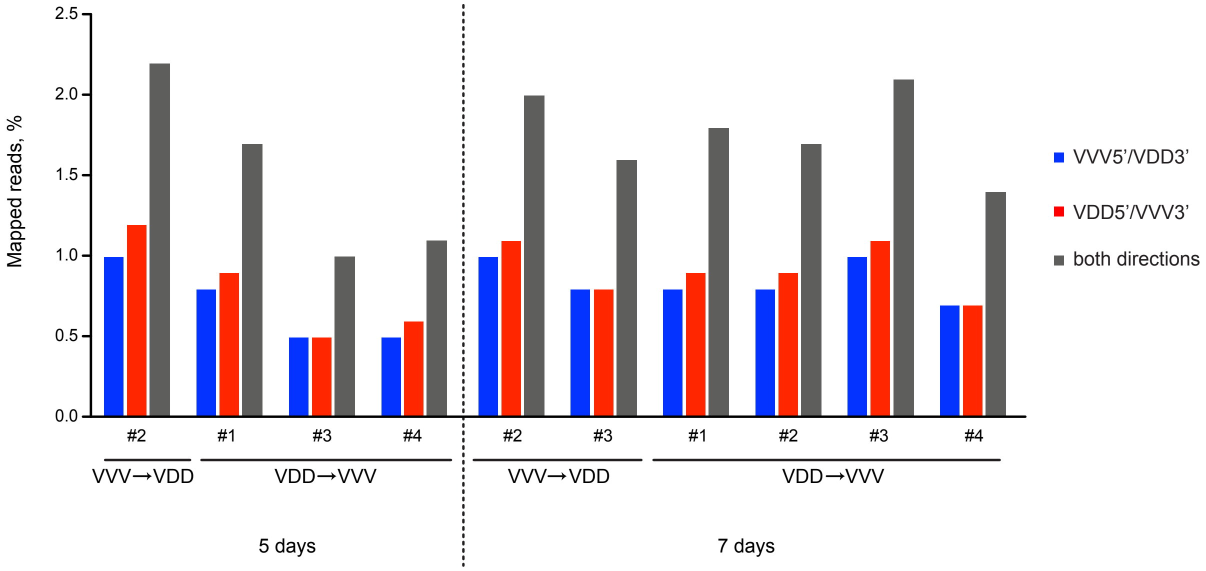


**Figure S9. Intertypic recombination reads in each of the honey bee pupae samples analysed using ViReMa and shown as a percentage of all mapped Illumina reads.** Blue and red bars represent amounts of recombinants with VVV5'/VDD3' and VDD5'/VVV3' directionality respectively, grey bars show the total amount of recombinants of both directionalities in each sample. Each cluster of bars corresponds to a single pupa sample with a sample number shown below the axis. "VVV→VDD" and "VDD→VVV" indicate samples from the corresponding superinfection injection groups and order of inoculation. 5 and 7 days refer to the incubation period of injected pupae after the superinfection injection.

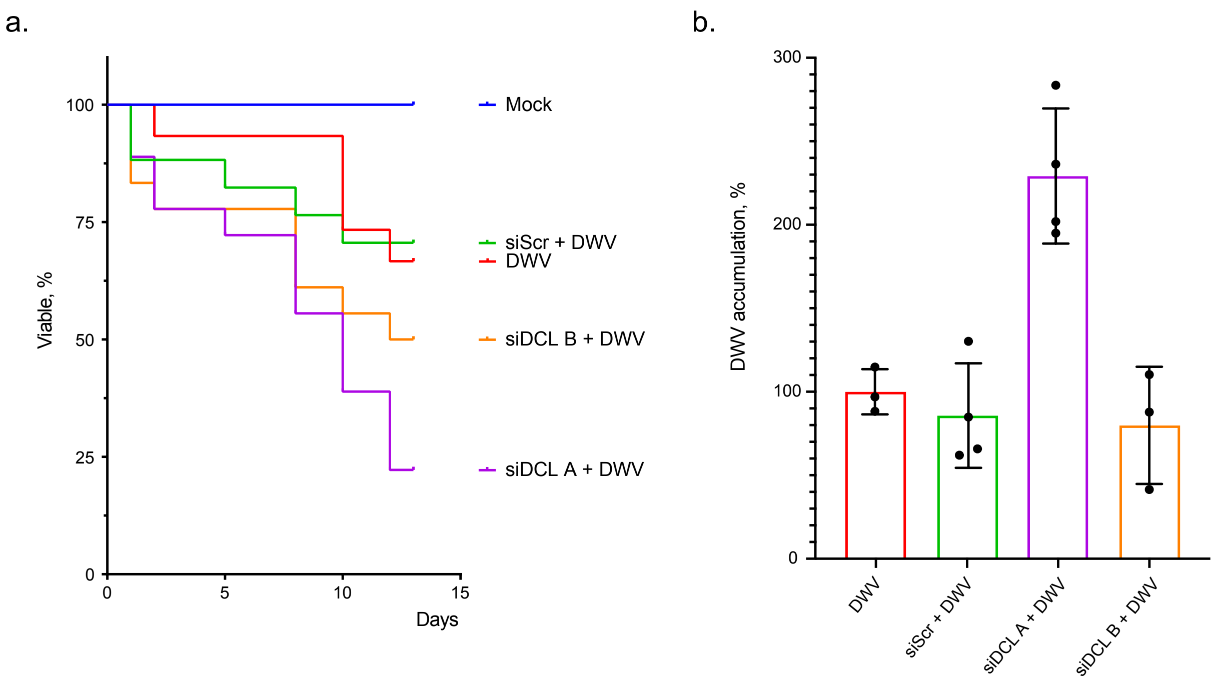


**Figure S10**: **Anti-Dicer siRNA treatment of honey bee larvae**. siRNA treatment was delivered in 5 µl of diet 3 times per day with 0.3-0.4 µg of siRNA supplied to each larva with each feeding and continued for 48 h after DWV inoculation (introduced together with 4th siRNA dose) or until pupation. “siDCL A” and “siDCL B” - larvae fed with specific siRNA targeting *Apis mellifera Dicer-like* mRNA (two different siRNA sequences were used), “siScr” - honey bee larvae fed with siRNA, which does not target any honey bee transcripts (control treatment), “DWV” - larvae fed with virus only (no siRNA treatment), “Mock” - non-treated control group. **a.** Survival time course of honey bee larvae fed with siRNA and infected with VVD DWV. First instar honey bee larvae were treated with siRNA introduced with feeding from day one to day five of the experiment. On the second day larvae were orally infected with $10^6$ GE of VVD DWV; n=16 in each treatment group. **b.** qPCR analysis of DWV accumulation in honey bee larvae fed with siRNA and orally infected with $10^5$ GE of VVD DWV. Larvae were analysed 48 h after DWV feeding (72 h after the start of the siRNA treatment). Mean virus level in the DWV only group is set as 100%, each dot corresponds to DWV level in an individual larva sample, error bars representing mean ±SD.

**Table S1. PCR primers and oligonucleotides used in this study.**

| Name | Sequence (5'-3') | Target | cDNA binding site | Product size | Reference |
|---|---|---|---|---|---|
| **DWV_RTPCR_F** | ATTGATCATTGTATGTTTACCTTCCCTTG | DWV | 4021-4049 | 1606 bp | [25] |
| **DWV_RTPCR_R** | GCACGTAAGAGCTCGCTGCATA | DWV | 5606-5627 | | [25] |
| **DWV_qPCR_F** | ATATCACTTGGCGACGCAAC | DWV | 4950-4969 | 176 bp | [25] |
| **DWV_qPCR_R** | CCAATCTTTAAATTGTTTCGGTTTTTGAGC | DWV | 5098-5126 | | [25] |
| **DWV-A (RdRp) F** | TGTCTTCATTAAAGCCACCTGGAA | DWV type A | 8654-8677 | 140 bp | [13] |
| **DWV-A (RdRp) R** | TTTCCTCATTAACTGTGTCGTTGAT | DWV type A | 8769-8793 | | [13] |
| **VDV (RdRp) F** | TATCTTCATTAAAACCGCCAGGCT | DWV type B | 8654-8677 | 140 bp | [13] |
| **VDV (RdRp) R** | CTTCCTCATTAACTGAGTTGTTGTC | DWV type B | 8769-8793 | | [13] |
| **DWV type A StPr F** | GTCAAGAAGCAGGCGAATGTA | DWV type A | 1502-1522 | 129 bp | *This study* |
| **DWV type A StPr R** | GCATAGGGGATCTAGAACACATAG | DWV type A | 1608-1630 | | *This study* |
| **DWV type B StPr F** | GAAAAGACGCGGGTGAGTTCG | DWV type B | 1502-1522 | 130 bp | *This study* |
| **DWV type B StPr R** | AACATTGGCGATCGATTACAAACG | DWV type B | 1608-1631 | | *This study* |
| **Apis mellifera actin F** | AGGAATGGAAGCTTGCGGTA | *Apis mellifera actin* | 919–938 | 181 bp | [9] |
| **Apis mellifera actin R** | AATTTTCATGGTGGATGGTGC | *Apis mellifera actin* | 1099–1079 | | [9] |
| **EGFP_qPCR_F** | GAGAAGCGCGATCACATGGT | EGFP | 639-659 | 189 bp | [25] |
| **VDD_VP2qPCR_RP** | AGATGTACTAGGATCTCGCTGAGTT | DWV | 1860-1884 | | [25] |
| **mCherry_qPCR_F** | CAAGTTGGACATCACCTCCCAC | mCherry | 606-627 | 163 bp | [25] |
| **VVV_VP2qPCR_RP** | TAATTCAACTTCACCTTCGCCATCTG | DWV | 1808-1833 | | [25] |
| **DWV FG FP4** | GCGAATTACGGTGCAACTAAC | DWV | 19-39 | 10 068 bp | [38] |
| **DWV FG RP1** | TACGCGAGTAACACCTAAC | DWV | 10 076-10 094 | | [38] |
| **siScr1_FW** | GGATCCTAATACGACTCACTATAGTCTAGGACCTCTGATATC | - | | | *Jarosch et al, 2011* |
| **siScr1_RV** | AAGATATCAGAGGTCCTAGACTATAGTGAGTCGTATTAGGATCC | - | | | *Jarosch et al, 2011* |
| **siScr2_FW** | GGATCCTAATACGACTCACTATAGATATCAGAGGTCCTAGAC | - | | | *Jarosch et al, 2011* |
| **siScr2_RV** | AAGTCTAGGACCTCTGATATCTATAGTGAGTCGTATTAGGATCC | - | | | *Jarosch et al, 2011* |
| **siDCL A top aintisense** | GGATCCTAATACGACTCACTATAGACTATGTACATAACGTGC | *Apis mellifera Dicer-like* | | | *This study* |
| **siDCL A bottom aintisense** | AAGCACGTTATGTACATAGTCTATAGTGAGTCGTATTAGGATCC | *Apis mellifera Dicer-like* | | | *This study* |
| **siDCL A top sense** | GGATCCTAATACGACTCACTATAGCACGTTATGTACATAGTC | *Apis mellifera Dicer-like* | | | *This study* |
| **siDCL A bottom sense** | AAGACTATGTACATAACGTGCTATAGTGAGTCGTATTAGGATCC | *Apis mellifera Dicer-like* | | | *This study* |
| **siDCL B top aintisense** | GGATCCTAATACGACTCACTATAGATATACACCAATCAATGC | *Apis mellifera Dicer-like* | | | *This study* |
| **siDCL B bottom aintisense** | AAGCATTGATTGGTGTATATCTATAGTGAGTCGTATTAGGATCC | *Apis mellifera Dicer-like* | | | *This study* |
| **siDCL B top sense** | GGATCCTAATACGACTCACTATAGCATTGATTGGTGTATATC | *Apis mellifera Dicer-like* | | | *This study* |
| **siDCL B bottom sense** | AAGATATACACCAATCAATGCTATAGTGAGTCGTATTAGGATCC | *Apis mellifera Dicer-like* | | | *This study* |

**Table S2. Percentage of samples with visible EGFP expression in each group of pupae superinfected with $VDD_E$ and analysed intact by confocal microscopy.**

| Injection group | Head | | Wing | | Abdomen | |
|---|---|---|---|---|---|---|
| | 1 day | 4-5 days | 1 day | 4-5 days | 1 day | 4-5 days |
| **Mock→$VDD_E$** | **100%** | **100%** | **100%** | **100%** | **100%** | **100%** |
| **VVV→$VDD_E$** | 0% | **100%** | **40%** | **100%** | **100%** | **100%** |
| **VVD→$VDD_E$** | 0% | **25%** | 0% | 0% | 0% | **88%** |
| **VDD→$VDD_E$** | 0% | 0% | 0% | 0% | 0% | 0% |
| **DDD→$VDD_E$** | 0% | **25%** | 0% | 0% | 0% | **38%** |

**Table S3. Positions of the most frequently observed recombination junctions in VVV and VDD DWV superinfected pupae when recombining in both directions, from VVV5'/VDD3' predominantly or VDD5'/VVV3' only.**

| **Recombinant formed** | **Recombination point** | **Total mapped reads** |
|---|---|---|
| | **5' nucleotide** | |
| Both directions | 3452 | 600 |
| | 5007 | 1930 |
| | 5582 | 1179 |
| | 8060 | 789 |
| Predominantly VVV5'/VDD3' | 2516 | 246 |
| | 3932 | 257 |
| | 5021 | 326 |
| | 5646 | 266 |
| | 8405 | 242 |
| Only VDD5'/VVV3' | 4371 | 271 |
| | 4830 | 365 |
| | 4971 | 273 |
| | 6288 | 362 |
| | 8733 | 221 |

**Table S4. Genetic identity of RG DWV genomes.** DWV VVV, VVD, VDD, and DDD genomes were aligned with ClustalX and the genome length genetic identity (percentage) calculated, as shown in the white cells in the upper right half of the table. Aligned DWV genomes were subsequently analysed for the percentage identity of sequences in excess of 20 nucleotides, as shown in the black shaded cells of the table. For example, VVV and DDD share 85% genetic identity, of which only 34% is located in contiguous sequences of more than 20 nucleotides.

| Identity | VVV | VVD | VDD | DDD |
|---|---|---|---|---|
| VVV | 100 | 93 | 87 | 85 |
| VVD | 68 | 100 | 94 | 92 |
| VDD | 41 | 73 | 100 | 98 |
| DDD | 34 | 67 | 93 | 100 |

**Text S1. Full cDNA sequences of DDD, VVV$_{mC}$, and VVD$_{S}$ RG clones.** DDD and VVV$_{mC}$ constructs were obtained by modification of VDD (GenBank MT415949) and VVV (GenBank MT415952) cDNAs respectively, VVD$_{S}$ represents a *SalI*-tagged variant of VVD. Gene-synthesis insert homologous to 5'-UTR sequence of DWV A (reference sequence - DWV-A 1414, GenBank KU847397) is highlighted in grey, mCherry encoding sequence is shown in red with flanking inserts encoding protease cleavage site duplication shown in bold font, *SalI* restriction site in VVD$_{S}$ sequence is highlighted in yellow with nucleotides differing from the field virus sequence (GenBank HM067438.1) shown in bold.

>DDD

tttaaaattcgctatgggaggcgatttatgccttccatagcgaattacggtgcaactaacaattttagata
gtagccataaacagacattatagtagctcactacgtattgatcattttataatgacttgcgtagtatgaagcgcat
gcttgtagttgtaactatgttactttgcaagttggagcttactattttggattatgaatatgtgcacttagtgtctg
tatttatagtcgtttgtggttcaaggttttgtgttagtagtacacttatgtatgaatgtacctttagtatgaatgtt
atagaatgacaatatcgaaggaaaaatctttataaaatacaaaaatattgtttttattatttcgatatggtgtttta
tagagtagattgccatgtgaccgctcatagaagtccattatggtttatcaatcgaagttgaatgtatttataagaat
attatacttaattagtaatattagtagtccgtaactattatcatccttttacagtttgatgtgataatagaccactg
cagtatcgagtagagtttcgaatgcgtagtgcaatagtacaatcactgtcaccgaccatctatcgtaatgatagatc
tgtcggaaaccattatttatgaagtgactagcaatcatggattaaattagatggtattctagtttagaggtgattcg
gcgctgcggtgcgactgaaacttctaaattagcatgtcagattgtattatgaatgcgttagtagtaatttctgcgat
agagctgggacccctcagtctctcaggtattgtatgaggcgaaagtgtgaaagttttgtatgtatttttttatatgt
acgactgtatcgggaattcctttagcaagaatccttttaatacagtataatctgtgctacggtacgttatgttcgca
gggcacccgttaatgtctcatagcccagacgatggcggatggaaagacatcatattttattttaatgctgtctttat
tgctgatttattttgctgtttttatttgctattttatatttgctaattttcattattgcgaaatatattatattgct
atttttattatatacgctagattcaattttattctttctatattttcaatttaattttgatttcgaaggtaaatata
tataattgattattaaaaatggcctttagttgtggaactctttcttactctgccgtcgcccaagctccgtctgtcgc
ctatgcacctcgtacatgggaagttgatgaagctaggcggcgccgagtcattaaacgtttggcgctggagcaagaac
gtattcgtaacgttcttgacgttgccgtctatgaccaggcgacatgggaacaggaggacgcgcgcgataatgagttc
ctaacggaacaattaaacaatttatatactatttattcgatcgctgaacgttgtacgcgtcggcctatcaaagagta
ctctcctatatcagtttcgaataggtttgctccactggaatccctcaaggtcgaggtcggtcaagaagcaggcgaat

gtatatttaagaaacctaaatatacgcgcgtttgcaagaaagtgaagcgtgttgcaactcgcttcgttcgtgaaaaa
gttgttcgtcctatgtgttctagatcccctatgctattatttaagcttaagaaaattatttatgatttgcatttata
tagattaagaaaacagattaggatgttgagacgtcaaaaacagcgcgattatgagttagagtgtgtcactaatctgt
tacaattatcgaatccagtgcaggcaaaaccagagatggataaccctaatccaggacctgatggcgagggtgaagtt
gaattagaaaaggatagcaatgttgttttaacaactcagcgagatcctagtacatctattccagcgccggtgagcgt
aaaatggagtagatggactagtaatgacgtagtagatgattatgccacaatcacatctcgatggtatcagattgctg
aatttgtttggtcgaaggatgatccatttgataaggagttagcacgtttaattttgcctcgtgctttgttatctagt
atagaggctaattctgatgctatatgtgatgtgcctaatactatcccatttaaggtacacgcatattggcgaggcga
tatggaagttagagttcaaattaattcaaataaattccaagttggtcaattacaagctacttggtattattcggatc
atgagaatttgaatatatcgtctaagagaagcgtttatggattttcacaaatggatcatgctttgattagtgcgtca
gcaagtaatgaagcaaaattagttattccatataagcatgtttatccatttttaccgacaagaattgtgccagattg
gactactggcattttagatatgggtgctttgaacattcgtgtaattgctcccttacggatgagtgctactggtccaa
ctacctgtaatgtcgtcgtgtttattaaattaaataacagcgagtttacagggacttcttctggtaagttttatgcg
agccaaatcagggcaaaacctgagatggatcgtatattaaatttggcagagggattgttgaataacacgattggtgg
taataatatggataatccttcttatcaacaatctcctcgtcattttgtcccgactggtatgcacagcttagctttag
gtactaatttagttgaaccattacatgcattacgtttggatgcagccggtacgacacaacatcctgtaggttgtgct
ccggatgaagatatgactgtatcctccattgcatctcgatatggactaattagacgggtacaatggaagaaagatca
tgctaaaggatcacttttgttacaattagatgctgatccatttgtggagcaaagaattgaaggtacgaatccaatat
ctttgtattggttcgcacccgtgggtgtagtatctagtatgtttatgcaatggcgcggttcattagaatataggttt
gatattatagcatcccaatttcatactggtaggttaattgtaggttatgtgcccggtttgacagcatctttgcaact
tcaaatggactatatgaaattgaagtcatcgagttatgtagtatttgatttacaagaaagtaatagcttcacttttg
aggtgccatatgtttcatatagaccatggtgggtgcgtaaatatggtggcaattatttaccctcgtcaactgacgct
cctagtacattatttatgtatgtgcaggttccgttgatacctatggaagctgtttcagatactattgatatcaatgt
gtacgtacggggcggtagttcatttgaagtttgtgttccagtccaacctagtttaggtttgaattggaatacagact
ttattttacgtaatgacgaagaatacagggctaagacaggttatgcaccatattatgctggagtgtggcatagcttc
aataatagtaattctcttgtttttaggtggggatctgcttccgatcaaattgctcagtggccgacaatttcagtacc
aagaggtgagctagctttcttacgaattaaggatggaaagcaagctgctgtaggaactcaaccttggcgtacgatgg
ttgtttggccttctggtcatggttataatattggtatacctacgtataatgctgaacgagctcgccagcttgcacaa
cacttatatggtggtggatcattaactgatgagaaggccaaacaattatttgttcctgctaatcaacaaggacctgg

taaggtaagtaatggaaatccggtatgggaagtcatgcgtgcaccattggcaacacagcgtgcgcatattcaagatt

ttgaatttattgaagctattccagaaggagaggagtctcgtaatactacagtcttggatacgaccactactttacag

tcgagtggatttggtcgcgccttctttggagaagcttttaatgaccttaaaacgttaatgcgacgatatcaattata

tggtcaattattattgtccgttactacggataaggatattgatcattgtatgtttaccttcccttgtttaccacaag

ggttagcgttagacattggttctgctggctctccacatgaaatctttaatagatgtcgtgatggtattataccatta

attgcatctggatatagattttatagaggagatttgcgttataagattgtttttccaagtaatgttaatagcaacat

ttgggtacaacatcgaccggatcgtagactggaaggatggtccgcggctaagattgtaaattgtgatgctgtgtcta

ctggtcaaggggtgtataatcatggttatgctagtcacattcaaatcacgcgtgtaaataatgttatagaattggaa

gttccattttataatgctacttgttataattatttacaggcgtttaatgcgtctagcgctgcatctagttatgcagt

atctttaggagaaatatcggttggttttcaagctacaagtgatgatattgcatctattgttaacaaacctgttacta

tttattatagtattggagatggtatgcaattttctcagtgggttggatatcaaccgatgatgatcctagatcagctt

cctgcaccagtagtaagggccgtgcctgagggccctattgcgaagattaaaaacttcttccatcaaacagccgacga

agttagagaagctcaggcagcaaagatgcgtgaagatatgggtatggttgtccaagatgttattggagaacttagcc

aggccataccggatcttcaacaaccggaggttcaagcaaatgtcttctcactggtgtctcagttagtgcatgctatt

ataggtactagtttgaagacagttgcttgggcgattgtttcgatttttgtgaccctaggtttgattggacgtgaaat

gatgcattcagtcataactgtagttaagcggttattagaaaaatatcacttggcgacgcaaccccaggaatccgcca

attcaggtacggttatttccgctgttccagaagcacctaatgctgaagcagaggaggccagtgcctgggtatccatt

atttataatggtgtgtgtaatatgttgaatgtagccgctcaaaaaccgaaacaatttaaagattgggtaaaattagc

taccgtagattttagtaataattgtagaggtagtaaccaggtatttgtatttttcaagaatacatttgaagtgttga

agaaaatgtggggttatgtattttgtcaaagtaatcctgcagcgcgtttgttgaaagctgtgaatgacgagcctgag

attttgaaagcatgggtgaaggagtgtctgtatttggatgatcctaaattcagaatgcgtcgagcgcatgatcaaga

gtatatcgagagagtgtttgcggcacattcatatggacaaattttgctacatgatttaactgctgaaatgaatcaat

cacgaaatttgagtgtgtttacacgtgtgtatgatcaaatttcaaaattgaaaaccgatcttatggaaatgggatca

aatccatatataaggcgtgaatgttttacgatatgcatgtgtggtgcatctggaattggaaaatcatatttgaccga

ttctttatgcagcgagctcttacgtgcgagtcgtactcctgtgacaacaggcataaaatgtgttgttaatccattat

ctgattattgggatcaatgtgattttcagcctgttttgtgcgttgacgatatgtggagtgttgaaacatctactacg

ctcgataagcagttgaatatgcttttccaggttcattctcctatcgtactttctcctcctaaagctgatttagaagg

taagaaaatgcgatataacccggaaatattcatatacaatacgaataaacctttcccgaggtttgaccgtattgcta

tggaagctatttatcggcgtagaaatgttttgattgaatgtaaagcgagtgaagagaagaagcgaggatgtaagcat

tgtgagaatgatattcctattgctgaatgtagtcctaagatgttgcaagattttcatcatatcaaatttaggtatgc

acatgatgtatgtaattctgagaccacatggtctgaatggatgacgtatagtgaatttcttgaatggataactcctg

tgtatatggctaatcgtcgtaaggcgaatgaatcgtttaagatgcgtgtggatgaaatgcaaatgttacgtatggat

gaaccattagaaggtgataatattctcaataagtatgttgaagttaatcagcgcttagtggaggaaatgaaggcatt

taaggaacgcacactatggtcagatttacatcgcgtaggtgcggaaattagtgcgtcagttaagaaagctttaccaa

ccatttccataaccgaaaaactaccacattggactgttcaatgtggcattgctaaacctgagatggatcatgcttat

gaggttatgagttcgtatgcagctggaatgaatgcggagattgaagcgcatgaacaagtccggcgttcatcagtgga

atgtcaatatgcagagcctcaagcttcaagaaatcctgatgatgaagggccaaccatagatgaagaacttatgggcg

acactgaatttacatcacaggctttagaacgtcttgtggatgaaggttatataactggaaaacagaagaaatatata

gctacgtggtgtagtaagcgtcgtgaacatactgctgactttgatcttgtctggactgataatttgcgtgtgttaag

tgcgtatgcgcatgaacgctcatcttcaactcggctttctacggatgatgtcaagttatataaaacaattagcatgt

tacatcaaaagtatgataccacagagtgtgctaaatgtcaacattggtatgctccgttgactgatatctatgttgat

gataagaaattgttttggtgtcagaaagagaaaaagacacttatcgatgtccgcaaattgtcgaaagaagatgtgac

tgttcaatcaaaattgattaatttatctgttccttgtggtgaagtgtgtatgttacattcaaaatatttcaattatc

ttttccacaaagcatggttgtttgagaacccaacttggcgcctaatatataatggtaccaagaagggtatgcctgag

tactttatgaattgtgtggatgaaatttcattagattccaaatttggtaaagtgaaagtatggttgcaagcgatcat

tgataagtatttaactcgtcccgtgaaaatgattcgtgattttcttttcaagtggtggccgcaagttgcgtatgtgt

tgagcttgctaggtataattggtataactgcgtatgaaatgagaaatccgaaaccaacttctgaggaattagctgat

cattatgtgaataggcattgtagctctgattttggtcaccaggactggcatcacctcaaggattgaaatatagtga

agcagtaacagcaaaggcacctagaatccatagattgccagtgactactaagcctcagggatcaactcaacaagtag

acgctgctgtgaataaaattttacagaacatggtttacattggtgttgttttcccgaaagtgcctggtagtaagtgg

cgagatattaattttaggtgtcttatgcttcataataggcaatgtttaatgttgaggcattatattgagtcaactgc

cgcctttcctgaggggaccaagtactattttaagtatattcataatcaagagactagaatgtctggtgatatttctg

gtattgaaattgatttgttgaatttacctagattgtattatggtggtctcgcgggagaggagtcatttgatagcaat

attgtgcttgtgactatgcctaatcgtattcctgagtgtaagagcattattaaatttattgcgtcacataatgaaca

tatacgtgctcagaatgatggagtgttagtaactggcgaccatactcagctattggctttcgagaataataataaga

ctccaataagtatcaacgctgatggtttgtatgaggttatacttcaaggagtatatacttatccataccatggcgat

ggtgtttgtggttccatattgctgtctcggaatttacaacggccgattataggtatccatgttgctggtactgaagg

attgcatggctttggagtcgctgaaccactggtacatgaaatgttcaccggtaaagcaatcgagagtgaaagagagc

cgtatgatcgtgtgtatgaacttccgttgcgtgaattagatgaatctgatattggtttagatactgatttatatccg
attggtagagtggatgcaaagttagctcatgctcaaagcccttctactgggatcaaaaagacgcttatccatggaac
atttgatgtaaggactgaaccaaatccgatgtcgtcacgtgatccaagaatagcgccgcatgatcctttgaagttag
ggtgtgaaaagcatggtatgccttgttcaccgtttaataggaaacatctggaattagcgacaaatcatttgaaagaa
aaattagtttcagtagttaaaccaataaatggttgcaagattagaagtttgcaagatgctgtatgtggtgtgcctgg
tttagatgggtttgattcgatatcttggaatactagtgctggttttcctttgtcttcattaaagccacctggaacat
ccggtaagcgatggttgtttgacattgagctgcaagactcgggatgttatctcctgcgtggaatgcgtcccgaactt
gagattcaattatcaacgacacagttaatgaggaaaaagggaataaaacctcacactatattcacggattgtttgaa
agatacttgtttgcctgttgaaaaatgtagaatacctggtaagactagaatatttagcataagtccggtgcagttta
ccataccgtttcgacagtattatttagactttatggcatcctatcgagctgcacgacttaatgctgagcatggtatt
ggtattgatgttaacagcttagagtggacaaatttggcaacaaggttgtctaagtatggcactcacatcgtgacagg
agactataagaattttggtcctgggttagattccgatgttgcagcttcagcgttcgaaattattatcgactgggtat
tacattacactgaagaagataataaagacgaaatgaagcgagtaatgtggaccatggcgcaagagatcttagcgcct
agtcatctatgtcgcgacttggtgtaccgagtaccttgcggaattccatcaggttctccaataacggacatattgaa
tacaatttcaaattgtttgttaattaggttagcctggttaggtattactgatttgcctttgtccgagttctctcaaa
atgttgttcttgtttgttatggtgatgatcttatcatgaatgttagtgataacatgattgataaatttaatgctgtg
acaatagggaaattcttttcacaatataagatggaatttacggatcaggacaaatcaggaaatactgtgaagtggcg
gacgttacagactgctactttcttgaagcatgggtttttaaaacatccaactagacctgtgtttctggctaacctag
acaaggtttcggtagaaggaacgacgaattggacccatgctcgaggattgggtcgtcgtacagcaaccatagagaat
gctaagcaagcgctagagttagcattcggatggggtccagaatactttaactatgtcagaaatactattaaaatggc
ttttgacaagttgggtatttatgaagaccttatcacatgggaagaaatggatgttagatgttatgctagcgcgtagt
atttaattttgaatacttattagttttaattttattttaggttattggaattgagggaagtaccacccccccaagacc
ttcgttttaaatctactaaaaggagtgaacctatatataagagtctaacgacagagtggatcagaccaccatcttta
gcttatatatgggaaaggttgagttgcctctaaagactcagctccatagtagagtagttttaattacgattaaagtg
gtactctaggttaggtgttactcgcgtattatcaactagtggtaatgcgtcctaatttagtatagttttaaccata
atagtaaaaaaaaaaaaaaaaaaaaaaaaaa

>VVV-mCherry ($VVV_{mC}$)

tttaaaattcgctatgggaggcgatttatgccttccatagcgaattacggtgcaactaacaattttagata
gtagccatgaacaaacattatgattactcactacgtattgatcatttttttcaatggcttgcgtagcatgaagcgcat

gcttgtagttataactatgttattttgcaagttggagataattgtattggattatggatgcgtgcactaagtgtcta

catctatagtcgtttgtggttcaagtttttgtgttagtagtacaatcttgaagaatgtaagtatcgtatgaatgata

tttgaatgacaacactgaagtataaaatatataaaatccaaaaatatttttaatcttattcagtgtagtgtttgata

gagtagaatgccatgtgaccgctcaaagaagtccattatggtatatcattcgaagtcgaatacttgtgtatagttat

tgtattttattagtaatattagtagtccgtaactatcataatcctattatagtttgattatatgatagaccactgca

gtatcgagtagagtttagaaagagtagtgcaatagtaagatcactgtcaccgaccactcattgtaatagtgaggttt

gtcggaaaccagttattgtgcagcgactagcaatcgtgaatcaatatagttggtattctaaatatgagacgattcgg

cgattttattgcgactgaaatttcatatttagcatgtcaggtcttattatgaatgctcgagtatttatttctgcggt

agagtagggacccctctatctctcaggtactgtatgaggcgaaagtgtgaaagtaacttatgtctctatacataagt

gactgtatcgggatttcctttggcaagaatccttttaatacagtataatttatgctacggtacgttacgttcgcagg

gcacccgttaatgtcacatagcccagacgatgacgaatggaaagacattactttttattttaatgctacgattattg

ctgttttattttgctgtttttatttgctattatattttgctattttcattattgctaaatatatttctttgctattt

ttgctttatatattagattcaattctttttattttatattttcaatttgattttgattttgaaggtaaatatatata

aaaatggcatttagttgtggaactctttcttatgctgctgttgcccaagctccctctgtagctcatgctccccgtag

ttgggagattgatgaagctaggcgtcgacgcgttatcaagcgtttggcgttggaacaggaacggattcgaaacgttc

tcgacgtcactgtgtatgatcatacaacgtgggagcaagaggatgcgcgtgataatgagttccttacggaacaattg

aataatttatatacgatatattctatagctgaaagatgtacccgccggcctgttcaagaacatgtccccatttcaat

cagtaatagatattcccctttagaatcccttaagattgaggtaggaaaagacgcgggtgagttcgtatttaagaaac

ccaaatatacaaagatttgtaagaaagtgaaacgggtggcatcaaaatttgtgcgcgagaaagttgttaggcccgtt

tgtaatcgatcgccaatgttattatttaaaattaagaaagtaatatatgatttacatttgtatcggttacggaaaca

agttcggcttctcagacgcgaaaaacagcgtgaatatgagttagagtgtgttactagtttgctacagctatctaatc

ctgtttcagctaaacctgag**atggacaatcctatggtgagcaagggcgaggaggataacatggccatcatcaaggag**

**ttcatgcgcttcaaggtgcacatggagggctccgtgaacggccacgagttcgagatcgagggcgagggcgagggccg**

**cccctacgagggcacccagaccgccaagctgaaggtgaccaagggtggccccctgcccttcgcctgggacatcctgt**

**cccctcagttcatgtacggctccaaggcctacgtgaagcaccccgccgacatccccgactacttgaagctgtccttc**

**cccgagggcttcaagtgggagcgcgtgatgaacttcgaggacggcggcgtggtgaccgtgacccaggactcctccct**

**gcaggacggcgagttcatctacaaggtgaagctgcgcggcaccaacttcccctccgacggccccgtaatgcagaaga**

**agaccatgggctgggaggcctcctccgagcggatgtaccccgaggacggcgccctgaagggcgagatcaagcagagg**

**ctgaagctgaaggacggcggccactacgacgctgaggtcaagaccacctacaaggccaagaagcccgtgcagctgcc**

**cggcgcctacaacgtcaacatcaagttggacatcacctcccacaacgaggactacaccatcgtggaacagtacgaac**

**gcgccgagggccgccactccaccggcggcatggatgaattgtacaaagctaaacctgag**atggacaatcctaatcct

ggtccagatggcgaaggtgaagttgaattagaaaaagatagtaatgtagtattaactacacaacgtgatccgagtac

ttctattcctgctccaactagtgtgaagtggagtagatggactagtaatgatgttgtggatgattatgccactataa

cttcgcgttggtatcagattgccgaatttgtatggtcaaaggatgatccatttgataaggaattggcgcgtttaatt

ttacctcgagctttgttatctagtattgaggctaattctgacgctatttgtgatgtacctaatactattccgtttaa

ggtacatgcatattggcgtggagatatggaagttcgagtgcagattaactcgaataaattccaggttggtcaattgc

aggcaacttggtactattcggatcatgaaaatttgaatatccagacgaagcgaagtgtgtatggttttcgcatatg

gatcatgctttgattagcgcatcagcgagtaatgaagcaaaattaatgataccttttaaacatgtatatccattctt

accaacgcgtgtcgttcctgattggacaactggtattcttgatatgggtaccttaaatattcgtgtaattgctccac

tacgtatgagtgcgacgggaccaaccacttgtaatgttgtagtatttattaagttaaataatagtgaattcactggt

acttcttctggtaagttttacgcgaatcaaatcagggcaaaacctgaaatggaccgtgtgttaaatttggcagaagg

attactaaataatactgtaggtggttgtaatatggataatccgtcatatcagcaatctccgcgtcattttgttccta

ctggtatgcatagtttagctttaggcactaatttagtagagcctttgcatgcattacgattagatgcatcaggtaca

acacaacatccagttgggtgtgcgcctgatgaagatatgactgtatcttccattgcatcacgatatggtttaattcg

ccaagtgcaatggaagaaagaccatgcgaaaggatcattattattacaacttgacgctgatcctttcgttgaacaga

aaattgagggaaccaatccaatttctttgtattggtttgctccagttggagtcgtatctagtatgtttatgcaatgg

agaggttctttagaatatagatttgatattatagcttcccaatttcatacgggtaggttaattgtaggttatgttcc

tggactgacagcttctttacaacgtcaaatggactatatgaaattgaaatcatctagttatgtggtgtttgatttac

aggaaagtaatagttttacgtttgaagtgccctatgtgtcatacagaccgtggtgggtgcgtaagtatggtggtaat

tatctgccatcttctactgatgcgcctagcacactgtttatgtatgtacaagtaccattgatacctatggaagctgt

ttctgatactatagatatcaatgtgtatgtgcgtggtggcagttcgtttgaggtttgtgttccagtccaacctagtt

taggtttgaactggaatacagatttcatattacgtaatgatgaagagtaccgcgcaaagaatggatatgcaccatat

tatgctggtgtgtggcatagcttcaataatagcaattcgcttgtttttagatggggttcggcttcagatcaaattgc

tcaatggccaacaataacagtgcctcgaggagagttggcattcttgcgtatccgcgatgctaagcaagctgctgtag

gaacgcaaccttggcgtactatggtcgtttggccttcaggtcatggatataatattggaataccaacttataatgct

gaacgagcaagacaacttgctcagcatttgtatggtggtgggtctttgacagatgaaaaggctaagcaattatttgt

gcctgctaaccagcaaggacccggcaaagtaagtaatggtaaccctgtctgggaagtaatgcgcgcgcctcttgcaa

ctcagcaagcgcatatacaagattttgaatttgttgaagctgttccagaaggcgaagaatcacgcaacactacggtg

ctagatacgacaataacgttacagtctagcggatttggtcgcgctttcttcggtgaggcatttaacgatcttaagac

gttaatgcgccgataccaattatatggtcaattattgttatccgttactacggataaggatattgatcattgtatgt

ttaccttcccttgtttacctcaagggctagcgttagatataggttcggctggatctcctcatgaaatatttaatcgc

tgccgtgatggtatcattccattgatagcgtcagggtatcggttttatcgaggcgatttacggtttaaaattgtttt

cccaagtaacgttaatagcaatatttgggtacaacaccgaccagatcgtagactgaaaggatggtctgaagcgaaaa

tagtaaactgtgatgctgtatctactggacaaggcgtttataatcatggatatgctagtcatattcagattacgcgt

gtaaataatgttatagaattggaagtcccgttttataacgctacgtgctataattatttgcaagcgtttaacccatc

tagtgcagcgtcaagttatgccgtttcgctcggagagatttcggttggttttcaagctactagcgatgacattgcag

ccatagttaataaacctgtaactatatattacagtattggcgatggtatgcagttttcgcagtgggttggttatcaa

ccaatgatgattctagatcaattgccagcaccagtagttagggctgtgcctgagggccctatagcgaagataaagaa

ctttttccatcaaacggcagatgaagttcgagaagctcaggccgcaaagatgcgtgaagatatgggtatagtagtcc

aagacgttataggagagttaagtcaggctatacccgatcttcaacaaccggaagttcaagcgaatgtttttttctctg

gtgtcacagttagtgcatgctatcatcggtactagtcttaagacagttgcttgggcgattgtttcgatttttgtaac

cctaggtttgattggacgtgaaatgatgcattcagtcataactgtagttaagcggttattagaaaaatatcacttgg

cgacgcaaccccaggaatccgccaattcaggtacggttatttccgctattccagaagcacccaatgctgaagcagag

gaggccagtgcctgggtatccattatttataatggtgtgtgtaatatgttgaatgtagccgctcaaaaaccgaaaca

atttaaagattgggtaaaattagctaccgtagattttagtaataattgtagaggtagtaatcaggtatttgtgtttt

tcaagaatacgtttgaagtgttgaagaaaatgtggggttatgtgttttgtcagagtaatcctgcagcgcgactcttg

aaagcagtgaatgatgaacctgagattttaaaagcgtgggttaaagaatgtctgtatttagatgatcctaaatttag

aatgcgacgtgcgcatgatcaagagtatattgagagagtgtttgcggcccattcgtatggacaaattttattgcatg

acttaacggctgaaatgaatcaatcgcgtaatttaagtgtgtttacgagagtgtatgatcaaatatctaaattgaag

acggatctcatggaaatgggatcaaacccatatatcaggcgtgaatgctttacgatttgtatgtgtggtgcatctgg

aattggtaagtcttatttaactgattctttatgcagcgagctcttacgtgcgagtcgtactccagtgacaacgggca

ttaagtgtgtcgtgaaccctttgtctgattattgggatcagtgtgattttcagcccgttttatgtgttgatgacatg

tggagtgttgaaacgtctactacgctcgataaacagttaaatatgctatttcaggttcattcaccaattgtactttc

acctcctaaagctgatttagaaggtaagaaaatgcgttataatcctgaaatattcatatataatacgaataaacctt

ttccgaggtttgatcgtatagctatggaagctatttatcgacgtagaaacgttttaattgaatgtaaggctaatgaa

gagaagaagcgtggatgtaaacattgtgagaataatatacccattgctgaatgtagtccaaaaattttgaaagattt

tcatcacattaaatttcgttatgctcatgatgtgtgtaattctgaaacgacgtggtctgagtggatgtcgtataatg

aatttttggaatggattactcctgtatatatggctaatcgacgtaaagcaaatgaatcgtttaagatgcgtgttgat

gaaatgcaaatgttgcgtatggatgagcccttggaaggcgataatattttaaataagtatgttgaagttaatcagcg

cttagttgaggaaatgaaagcttttaaagagcgaaccctctgggctgatttacaacgtgttggctcagagattagta

cttcagttaagaaagcattaccaactatttccattactgagaagctaccacattggactatccaatgtggcatagct

aagcctgaaatggatcatgcttatgaagttatgagttcatatgcagcaggaatgaacgcagaaattgaagcgcatga

acaagttcgtcgttcttctttggaatgtcagtgtattgagccttcaacttcaagacctctggatgaagagggtccta

ctatcgacgaggaattacttggcgaagtagaatttacttcttcagctttggagcgtttggttgatgaggggtatatt

actggtaaacaaaagaagtacatggcaacttggtgtacgaaacgaagagagcatgtatccgattttgatttagtatg

gacggataatttgcgtgttttgagtgcgtatgtccacgagcgttctacatctacgcgtttatctaccgatgatgtta

aattatttaagacgattagtatgttacatcagaggtatgataccactgattgtgcaaaatgccaacattggtatgca

ccattaacagctatttatgttgatgatagaaagctattttggtgccagaaggagactaagactttgatagatgttcg

taaattgtcgaaagaggacgttacagtccaatcgaaattaattaacttatcggttccgtgcggtgatgtatgtatgt

tacattctaagtactttaattatttattccataaagcgtggttgtttgaaaatccaacatggcgtttaatatataat

ggtactaagaaaggtatgcctgagtatttcatgaattgcgtggatgaaatttcattagattcaaaattttgtaaagt

aaaggtttggcttcaagcaattattgataaatatttgactcgtccagtgaaaatgattcgtgactttctatttaaat

ggtggccgcaagtagcatacgtgttaagtttgttaggtataattggtataactgcgtatgagatgcgtaatcctaaa

tcaacagcagaagacttggctgagcactatgttaataggcattgtagttcagattttggtcaccaggtatggcgac

tcctcagggattaaaatatagtgaagcgataacagctaaagcgcctagaatccatagattgcccgttactactagac

ctcagggatcaacgcaacaagttgacgccgctgtgaataagattttgcagaatatggtgtatatcggtgttgtgttt

ccgaaagtgcctggtagtaagtggcgagatattaattttagatgtcttatgcttcataatcggcaatgtttgatgtt

gcggcattacattgagtcgacggctgcttttccggagggtaccaaatactattttaagtatattcataatcaagaaa

ctcgaatgtcaggtgatatatctggtattgagattgatttattgagtttacctagattgtattatggtggcttagct

ggggaagagtcgttcgatagcaatatagtgttagtaactatgccgaatagaattcctgagtgtaagagtattgtgaa

gtttatagcttcacatgctgaacatgctcgtgctcaaaatgatggtgtgttagttactggtgaacatactcagttat

tggcattcgagaataataataaaacacctataagtattaatgctgatggtttgtatgaggttatacttcaaggagta

tacacttatccataccatggtgatggtgtttgtgggtctatattattgtctcgtaatttacaacgaccgattatagg

gatccatgtagctggtactgaaggattacatggctttggtgttgctgaacctcttgttcatgagatgttcactggga

aagcaatagagagtgaaagggaaccgtatgatcgtgtgtatgaattacctttgcgtgaattagatgaatctgatata

ggtttagatactgatttatatcctataggaagagttgatgcgaaattagctcatgcccaaagtccttcaacaggaat

taaaaagacgcttattcatggtacttttgatgttcggactgaaccgaatccgatgtcatcacgagacccaagaatag

caccacatgatccgttgaagttagggtgtgagaaacatggtatgccatgttctccatttaatcgaaaacatttggaa

ttagcaacgactcatttaaaggagaagttaatttccgtagttaaacctataaacggatgcaagattagaagtttgca

agatgctgtgtgtggtgtaccaggtttggatggctttgattcaatatcctggaatactagtgctggttttcctttat

cttcattaaaaccgccaggctcttctggtaagcgatggttgtttgatattgaattacaagattcaggatgttatctt

ttgagagggatgagacctgaacttgagatacagttgacaacaactcagttaatgaggaagaagggaataaagcctca

cactatattcacggattgtttgaaagatacatgtttgcctgtggaaaaatgcagaatacctggtaagactagaatat

ttagtataagtcccgtccaatttacgattccattccgacaatactatctcgattttatggcgtcgtaccgtgccgct

agacttaatgctgagcatggaataggtatagacgtgaacagcttggaatggacaaacttggcaacaagtctgtcgaa

gtatggcacgcatattgtgacaggagattacaagaattttggtcctgggttagattctgatgttgccgcttcagctt

tcgaaattatcattgattgggtgttaaattacactgaagaagatgataaagacgaaatgaagcgtgtaatgtggact

atggctcaggaaatcttagctcctagtcacttatgtcgtgatttagtatatcgcgtaccatgcggtattccttctgg

atcaccaattacggacattttgaatactatttcgaattgtttgttaattcgattggcttggcaaggtattactgatt

tgcctttatccgaattttctagacatgtcgtgctagtttgttacggtgatgatctcatcatgaatgtaagtgatgag

atgatagataaattcaacgctgtaacaattggcgatttcttttcgcgatataagatggaatttacggatcaggataa

atctggaaatacagtgcggtggcgaactttacaaactgccacgtttttgaagcatgggttcttgaaacatccaacaa

gacccgtgtttctagccaatctggataaggtttctatagaaggaacaaccaattggacacatgctcgaggattgggt

cgtcgagtagcaaccattgagaatgctaaacaagcgctagagttggcattcggatggggtcccgaatactttaatca

tgttcggaataccattaaaatggcattcgacaagttaggtatttatgaagatcttatcacatgggaagaaatggatg

ttagatgttatgctagcgcgtagtatttaattttgaatacttattagttttaattttattttaggttattggaattg

agggaagtaccacccccaagaccttcgttttaaatctactaaaaggagtgaacctatatataagagtctaacgaca

gagtggatcagaccaccatctttagcttatatatgggaaaggttgagttgcctctaaagactcagctccatagtaga

gtagttttaattacgattaaagtggtactctaggttaggtgttactcgcgtattatcaactagtggtaatgcgtcct

aattttagtatagttttaaccataatagtaaaaaaaaaaaaaaaaaaaaaaaaaaa

>VVDs

tttaaaattcgctatgggaggcgatttatgccttccatagcgaattacggtgcaactaacaattttagata

gtagccatgaacaaacattatgattactcactacgtattgatcatttttttcaatggcttgcgtagcatgaagcgcat

gcttgtagttataactatgttattttgcaagttggagataattgtattggattatggatgcgtgcactaagtgtcta

catctatagtcgtttgtggttcaagtttttgtgttagtagtacaatcttgaagaatgtaagtatcgtatgaatgata

tttgaatgacaacactgaagtataaaatatataaaatccaaaaatatttttaatcttattcagtgtagtgtttgata
gagtagaatgccatgtgaccgctcaaagaagtccattatggtatatcattcgaagtcgaatacttgtgtatagttat
tgtattttattagtaatattagtagtccgtaactatcataatcctattatagtttgattatatgatagaccactgca
gtatcgagtagagtttagaaagagtagtgcaatagtaagatcactgtcaccgaccactcattgtaatagtgaggttt
gtcggaaaccagttattgtgcagcgactagcaatcgtgaatcaatatagttggtattctaaatatgagacgattcgg
cgattttattgcgactgaaatttcatatttagcatgtcaggtcttattatgaatgctcgagtatttatttctgcggt
agagtagggacccctctatctctcaggtactgtatgaggcgaaagtgtgaaagtaacttatgtctctatacataagt
gactgtatcgggatttcctttggcaagaatccttttaatacagtataatttatgctacggtacgttacgttcgcagg
gcacccgttaatgtcacatagcccagacgatgacgaatggaaagacattacttttattttaatgctacgattattg
ctgttttattttgctgtttttatttgctattatattttgctattttcattattgctaaatatatttctttgctattt
ttgctttatatattagattcaattctttttattttatattttcaatttgattttgattttgaaggtaaatatatata
aaaatggcatttagttgtggaactctttcttatgctgctgttgcccaagctccctctgtagctcatgctccccgtag
ttgggagattgatgaagctaggcgtcgacgcgttatcaagcgtttggcgttggaacaggaacggattcgaaacgttc
tcgacgtcactgtgtatgatcatacaacgtgggagcaagaggatgcgcgtgataatgagttccttacggaacaattg
aataatttatatacgatatattctatagctgaaagatgtacccgccggcctgttcaagaacatgtccccatttcaat
cagtaatagatattcccctttagaatcccttaagattgaggtaggaaaagacgcgggtgagttcgtatttaagaaac
ccaaatatacaaagatttgtaagaaagtgaaacgggtggcatcaaaatttgtgcgcgagaaagttgttaggcccgtt
tgtaatcgatcgccaatgttattatttaaaattaagaaagtaatatatgatttacatttgtatcggttacggaaaca
agttcggcttctcagacgcgaaaaacagcgtgaatatgagttagagtgtgttactagtttgctacagctatctaatc
ctgtttcagctaaacctgagatggacaatcctaatcctggtccagatggcgaaggtgaagttgaattagaaaaagat
agtaatgtagtattaactacacaacgtgatccgagtacttctattcctgctccaactagtgtgaagtggagtagatg
gactagtaatgatgttgtggatgattatgccactataacttcgcgttggtatcagattgccgaatttgtatggtcaa
aggatgatccatttgataaggaattggcgcgtttaattttacctcgagctttgttatctagtattgaggctaattct
gacgctatttgtgatgtacctaatactattccgtttaaggtacatgcatattggcgtggagatatggaagttcgagt
gcagattaactcgaataaattccaggttggtcaattgcaggcaacttggtactattcggatcatgaaaatttgaata
tccagacgaagcgaagtgtgtatggtttttcgcatatggatcatgctttgattagcgcatcagcgagtaatgaagca
aaattaatgatacctttttaaacatgtatatccattcttaccaacgcgtgtcgttcctgattggacaactggtattct
tgatatgggtaccttaaatattcgtgtaattgctccactacgtatgagtgcgacgggaccaaccacttgtaatgttg
tagtatttattaagttaaataatagtgaattcactggtacttcttctggtaagttttacgcgaatcaaatcagggca

aaacctgaaatggaccgtgtgttaaatttggcagaaggattactaaataatactgtaggtggttgtaatatggataa

tccgtcatatcagcaatctccgcgtcattttgttcctactggtatgcatagtttagctttaggcactaatttagtag

agcctttgcatgcattacgattagatgcatcaggtacaacacaacatccagttgggtgtgcgcctgatgaagatatg

actgtatcttccattgcatcacgatatggtttaattcgccaagtgcaatggaagaaagaccatgcgaaaggatcatt

attattacaacttgacgctgatcctttcgttgaacagaaaattgagggaaccaatccaatttctttgtattggtttg

ctccagttggagtcgtatctagtatgtttatgcaatggagaggttctttagaatatagatttgatattatagcttcc

caatttcatacgggtaggttaattgtaggttatgttcctggactgacagcttctttacaacgtcaaatggactatat

gaaattgaaatcatctagttatgtggtgtttgatttacaggaaagtaatagttttacgtttgaagtgccctatgtgt

catacagaccgtggtgggtgcgtaagtatggtggtaattatctgccatcttctactgatgcgcctagcacactgttt

atgtatgtacaagtaccattgatacctatggaagctgtttctgatactatagatatcaatgtgtatgtgcgtggtgg

cagttcgtttgaggtttgtgttccagtccaacctagtttaggtttgaactggaatacagatttcatattacgtaatg

atgaagagtaccgcgcaaagaatggatatgcaccatattatgctggtgtgtggcatagcttcaataatagcaattcg

cttgtttttagatggggttcggcttcagatcaaattgctcaatggccaacaataacagtgcctcgaggagagttggc

attcttgcgtatccgcgatgctaagcaagctgctgtaggaacgcaaccttggcgtactatggtcgtttggccttcag

gtcatggatataatattggaataccaacttataatgctgaacgagcaagacaacttgctcagcatttgtatggtggt

gggtctttgacagatgaaaaggctaagcaattatttgtgcctgctaaccagcaaggacccggcaaagtaagtaatgg

taaccctgtctgggaagtaatgcgcgcgcctcttgcaactcagcaagcgcatatacaagattttgaatttgttgaag

ctgttccagaaggcgaagaatcacgcaacactacggtgctagatacgacaataacgttacagtctagcggatttggt

cgcgctttcttcggtgaggcatttaacgatcttaagacgttaatgcgccgataccaattatatggtcaattattgtt

atccgttactacggataaggatattgatcattgtatgtttaccttcccttgtttacctcaagggctagcgttagata

taggttcggctggatctcctcatgaaatatttaatcgctgccgtgatggtatcattccattgatagcgtcagggtat

cggttttatcgaggcgatttacggtttaaaattgttttcccaagtaacgttaatagcaatatttgggtacaacaccg

accagatcgtagactgaaaggatggtctgaagcgaaaatagtaaactgtgatgctgtatctactggacaaggcgttt

ataatcatggatatgctagtcatattcagattacgcgtgtaaataatgttatagaattggaagtcccgttttataac

gctacgtgctataattatttgcaagcgtttaacccatctagtgcagcgtcaagttatgccgtttcgctcggagagat

ttcggttggttttcaagctactagcgatgacattgcagccatagttaataaacctgtaactatatattacagtattg

gcgatggtatgcagttttcgcagtgggttggttatcaaccaatgatgattctagatcaattgccagcaccagtagtt

agggctgtgcctgagggccctatagcgaagataaagaactttttccatcaaacggcagatgaagttcgagaagctca

ggccgcaaagatgcgtgaagatatgggtatagtagtccaagacgttataggagagttaagtcaggctatacccgatc

ttcaacaaccggaagttcaagcgaatgtttttctctggtgtcacagttagtgcatgctatcatcggtactagtctt

aagacagttgcttgggcgattgtttcgatttttgtaactttaggtttgattggacgtgaaatgatgcattcagtcat

aactgtagttaagcggttattagaaaaatatcacttggcgacgcaaccccaggaatccgccaattcaggtacggtta

tttccgctgttccagaagcacctaatgctgaagcagaggaggccagtgcctgggtatccattatttataatggtgtg

tgtaatatgttgaatgtagccgctcaaaaaccgaaacaatttaaagattgggtaaaattagctaccgt**c**ga**c**tttag

taataattgtagaggtagtaaccaggtatttgtatttttcaagaatacatttgaagtgttgaagaaaatgtggggtt

atgtattttgtcaaagtaatcctgcagcgcgtttgttgaaagctgtgaatgacgagcctgagattttgaaagcatgg

gtgaaggagtgtctgtatttggatgatcctaaattcagaatgcgtcgagcgcatgatcaagagtatatcgagagagt

gtttgcggcacattcatatggacaaattttgctacatgatttaactgctgaaatgaatcaatcacgaaatttgagtg

tgtttacacgtgtgtatgatcaaatttcaaaattgaaaaccgatcttatggaaatgggatcaaatccatatataagg

cgtgaatgttttacgatatgcatgtgtggtgcatctggaattggaaaatcatatttgaccgattctttatgcagcga

gctcttacgtgcgagtcgtactcctgtgacaacaggcataaaatgtgttgttaatccattatctgattattgggatc

aatgtgattttcagcctgttttgtgcgttgacgatatgtggagtgttgaaacatctactacgctcgataagcagttg

aatatgcttttccaggttcattctcctatcgtactttctcctcctaaagctgatttagaaggtaagaaaatgcgata

taacccggaaatattcatatacaatacgaataaacctttcccgaggtttgaccgtattgctatggaagctatttatc

ggcgtagaaatgttttgattgaatgtaaagcgagtgaagagaagaagcgaggatgtaagcattgtgagaatgatatt

cctattgctgaatgtagtcctaagatgttgcaagattttcatcatatcaaatttaggtatgcacatgatgtatgtaa

ttctgagaccacatggtctgaatggatgacgtatagtgaatttcttgaatggataactcctgtgtatatggctaatc

gtcgtaaggcgaatgaatcgtttaagatgcgtgtggatgaaatgcaaatgttacgtatggatgaaccattagaaggt

gataatattctcaataagtatgttgaagttaatcagcgcttagtggaggaaatgaaggcatttaaggaacgcacact

atggtcagatttacatcgcgtaggtgcggaaattagtgcgtcagttaagaaagctttaccaaccatttccataaccg

aaaaactaccacattggactgttcaatgtggcattgctaaacctgagatggatcatgcttatgaggttatgagttcg

tatgcagctggaatgaatgcggagattgaagcgcatgaacaagtccggcgttcatcagtggaatgtcaatatgcaga

gcctcaagcttcaagaaatcctgatgatgaagggccaaccatagatgaagaacttatgggcgacactgaatttacat

cacaggctttagaacgtcttgtggatgaaggttatataactggaaaacagaagaaatatatagctacgtggtgtagt

aagcgtcgtgaacatactgctgactttgatcttgtctggactgataatttgcgtgtgttaagtgcgtatgcgcatga

acgctcatcttcaactcggctttctacggatgatgtcaagttatataaaacaattagcatgttacatcaaaagtatg

ataccacagagtgtgctaaatgtcaacattggtatgctccgttgactgatatctatgttgatgataagaaattgttt

tggtgtcagaaagagaaaaagacacttatcgatgtccgcaaattgtcgaaagaagatgtgactgttcaatcaaaatt

gattaatttatctgttccttgtggtgaagtgtgtatgttacattcaaaatatttcaattatcttttccacaaagcat

ggttgtttgagaacccaacttggcgcctaatatataatggtaccaagaagggtatgcctgagtactttatgaattgt

gtggatgaaatttcattagattccaaatttggtaaagtgaaagtatggttgcaagcgatcattgataagtatttaac

tcgtcccgtgaaaatgattcgtgattttcttttcaagtggtggccgcaagttgcgtatgtgttgagcttgctaggta

taattggtataactgcgtatgaaatgagaaatccgaaaccaacttctgaggaattagctgatcattatgtgaatagg

cattgtagctctgattttggtcaccaggactggcatcacctcaaggattgaaatatagtgaagcagtaacagcaaa

ggcacctagaatccatagattgccagtgactactaagcctcagggatcaactcaacaagtagacgctgctgtgaata

aaattttacagaacatggtttacattggtgttgttttcccgaaagtgcctggtagtaagtggcgagatattaatttt

aggtgtcttatgcttcataataggcaatgtttaatgttgaggcattatattgagtcaactgccgcctttcctgaggg

gaccaagtactattttaagtatattcataatcaagagactagaatgtctggtgatatttctggtattgaaattgatt

tgttgaatttacctagattgtattatggtggtctcgcgggagaggagtcatttgatagcaatattgtgcttgtgact

atgcctaatcgtattcctgagtgtaagagcattattaaatttattgcgtcacataatgaacatatacgtgctcagaa

tgatggagtgttagtaactggcgaccatactcagctattggctttcgagaataataataagactccaataagtatca

acgctgatggtttgtatgaggttatacttcaaggagtatatacttatccataccatggcgatggtgtttgtggttcc

atattgctgtctcggaatttacaacggccgattataggtatccatgttgctggtactgaaggattgcatggctttgg

agtcgctgaaccactggtacatgaaatgttcaccggtaaagcaatcgagagtgaaagagagccgtatgatcgtgtgt

atgaacttccgttgcgtgaattagatgaatctgatattggtttagatactgatttatatccgattggtagagtggat

gcaaagttagctcatgctcaaagcccttctactgggatcaaaaagacgcttatccatggaacatttgatgtaaggac

tgaaccaaatccgatgtcgtcacgtgatccaagaatagcgccgcatgatcctttgaagttagggtgtgaaaagcatg

gtatgccttgttcaccgtttaataggaaacatctggaattagcgacaaatcatttgaaagaaaaattagtttcagta

gttaaaccaataaatggttgcaagattagaagtttgcaagatgctgtatgtggtgtgcctggtttagatgggtttga

ttcgatatcttggaatactagtgctggttttcctttgtcttcattaaagccacctggaacatccggtaagcgatggt

tgtttgacattgagctgcaagactcgggatgttatctcctgcgtggaatgcgtcccgaacttgagattcaattatca

acgacacagttaatgaggaaaaagggaataaaacctcacactatattcacggattgtttgaaagatacttgtttgcc

tgttgaaaaatgtagaatacctggtaagactagaatatttagcataagtccggtgcagtttaccataccgtttcgac

agtattatttagactttatggcatcctatcgagctgcacgacttaatgctgagcatggtattggtattgatgttaac

agcttagagtggacaaatttggcaacaaggttgtctaagtatggcactcacatcgtgacaggagactataagaattt

tggtcctgggttagattccgatgttgcagcttcagcgttcgaaattattatcgactgggtattacattacactgaag

aagataataaagacgaaatgaagcgagtaatgtggaccatggcgcaagagatcttagcgcctagtcatctatgtcgc

gacttggtgtaccgagtaccttgcggaattccatcaggttctccaataacggacatattgaatacaatttcaaattg

tttgttaattaggttagcctggttaggtattactgatttgcctttgtccgagttctctcaaaatgttgttcttgttt

gttatggtgatgatcttatcatgaatgttagtgataacatgattgataaatttaatgctgtgacaatagggaaattc

ttttcacaatataagatggaatttacggatcaggacaaatcaggaaatactgtgaagtggcggacgttacagactgc

tactttcttgaagcatgggtttttaaaacatccaactagacctgtgtttctggctaacctagacaaggtttcggtag

aaggaacgacgaattggacccatgctcgaggattgggtcgtcgtacagcaaccatagagaatgctaagcaagcgcta

gagttagcattcggatggggtccagaatactttaactatgtcagaaatactattaaaatggcttttgacaagttggg

tatttatgaagaccttatcacatgggaagaaatggatgttagatgttatgctagcgcgtagtatttaattttgaata

cttattagttttaattttattttaggttattggaattgagggaagtaccacccccccaagaccttcgttttaaatcta

ctaaaaggagtgaacctatatataagagtctaacgacagagtggatcagaccaccatctttagcttatatatgggaa

aggttgagttgcctctaaagactcagctccatagtagagtagttttaattacgattaaagtggtactctaggttagg

tgttactcgcgtattatcaactagtggtaatgcgtcctaattttagtatagttttaaccataatagtaaaaaaaaaa

aaaaaaaaaaaaaaaaa
